# Combined Use of Immune Checkpoint Inhibitors and Phytochemicals as a Novel Therapeutic Strategy against Cancer

**DOI:** 10.7150/jca.85966

**Published:** 2023-07-24

**Authors:** LingJie Luo, Caiji Lin, Pengfei Wang, Danli Cao, Yiru Lin, Wenxue Wang, Yufan Zhao, Yongwei Shi, Zixiang Gao, Xin Kang, Yuanyuan Zhang, Shuang Wang, Jiaxing Wang, Mengzhi Xu, Huidi Liu, Shu-Lin Liu

**Affiliations:** 1 Genomics Research Center (Key Laboratory of Gut Microbiota and Pharmacogenomics of Heilongjiang Province, State-Province Key Laboratory of Biomedicine-Pharmaceutics of China), College of Pharmacy, Harbin Medical University, Harbin, 150081, China.; 2 National Key Laboratory of Frigid Zone Cardiovascular Diseases (NKLFZCD) College of Pharmacy, Harbin Medical University, Harbin, 150081, China.; 3 Harbin Medical University-University of Calgary Cumming School of Medicine Centre for Infection and Genomics, Harbin Medical University, Harbin, 150081, China.; 4 Department of Biochemistry and Molecular Biology, University of Calgary, Calgary, T2N 4N1, Canada.; 5 Department of Microbiology, Immunology and Infectious Diseases, University of Calgary, Calgary, T2N 4N1, Canada.; 6 Division of Anti-Tumor Pharmacology, State Key Laboratory of Drug Research, Shanghai Institute of Materia Medica, Chinese Academy of Sciences, Shanghai, 201203, China.

**Keywords:** Immune checkpoint inhibitor, Phytochemicals, Combination therapy, Immune-related adverse events, Predictive biomarker, Gut microbiota

## Abstract

Immune checkpoint inhibitor (ICI) therapy has dramatically changed cancer treatment, opening novel opportunities to cure malignant diseases. To date, most prevalently targeted immune checkpoints are programmed cell death protein 1 (PD-1) and cytotoxic T-lymphocyte-associated antigen 4 (CTLA-4), with many others being under extensive investigations. However, according to available data, only a fraction of patients may respond to ICI therapy. Additionally, this therapy may cause severe adverse immune-related side effects, such as diarrhea, headache, muscle weakness, rash, hepatitis and leucopenia, although most of them are not fatal, they can affect the patient's treatment outcome and quality of life. On the other hand, growing evidence has shown that phytochemicals with anticancer effects may combine ICI therapy to augment the safety and effectiveness of the treatment against cancer while reducing the adverse side effects. In this review, we summarize the state of art in the various experiments and clinical application of ICIs plus phytochemicals, with a focus on their combined use as a novel therapeutic strategy to cure cancer.

## Introduction

The past decade has witnessed enormous advances on understanding the immune checkpoints regarding their roles to help cancer cells elude immune surveillance *via* decreasing immune cell activity and suppressing immune responses. In the tumor microenvironment (TME), cancer cells escape from immune functions through the expression of immune checkpoints such as programmed cell death ligand 1 (PD-L1), cytotoxic T-lymphocyte-associated antigen 4 (CTLA-4), T-cell immunoglobulin and mucin domain-containing molecule-3 (Tim-3), lymphocyte-activation gene 3 (LAG-3) and T cell immunoglobulin and ITIM domain (TIGIT) 1-5. Tumor cells can adopt normal physiologic immune checkpoints and hence disrupt the balance between tumor cell proliferation and immune-surveillance, ultimately escaping from the host elimination. To reactivate the suppressed immune system and restore the suppressed ability of immune cells to recognize and kill cancer cells, scientists have developed monoclonal antibodies (mAbs) as immune checkpoint inhibitors to block the immune checkpoint interaction between cancer cells and immune cells 6. To date, seven inhibitors of PD-L1/PD-1 or CTLA-4 have received FDA (U.S. Food and Drug Administration) approval for treating malignant tumors (7-20; see Table 1). However, to date only a subset of patients may respond to this novel therapy, whereas the vast majority of patients do not benefit or even experience multiple immune-related adverse events (irAEs), such as diarrhea, endocrine disorders, thyroid dysfunction or diabetes 21. The side effects can be rather devastating to the immune system or other physiological functions and may lead to exacerbation of the disease or even death of the patient, calling urgently for a solution for the ICI therapy to achieve greater efficacy and in the meantime minimize or even completely remove such unfavorable reactions.

Plants harbor a great diversity of chemicals, many of which may have biological properties to augment ICI efficacy or ameliorate ICI adverse effects. For example, phytochemicals derived from several plants show multiple activities in the human body such as anti-oxidation, immune-protection and anti-inflammation 22-24, which may improve the outcomes of cancer patients and may possibly be used in conjunction with immune checkpoint inhibitors. In fact, a large number of phytochemicals, such as curcumin, resveratrol and epigallocatechin-3-gallate (EGCG), show positive results in combination therapies with ICIs for cancer treatment (25-36; see Table 2). At the same time, phytochemicals also influence the diversity and abundance of intestinal microbiota that exist in a symbiotic relationship with the host. Previous studies have exhibited that some microbial metabolites are immunoprotective while others contribute to chronic inflammation and cancer promotion. Increased level of toxic microbial metabolites plays a role in the formation of tumor microenvironment (TME) because of imbalance of intestinal microbiota, so maybe we can regard the balance of gut microbes as indicators of the efficacy of cancer treatment 37. Additionally, predictive biomarkers such as immune cells, PD-L1 overexpression, neoantigens, genetic and epigenetic signatures may also help screen patients for those who may have high responses to ICI therapy. Investigations on biomarkers for patient selection to achieve maximum efficacy and in the meantime alleviate side effects are rapidly progressing 38.

In this review, we first summarize the currently known immune checkpoints and relevant FDA-approved blockades, briefly discuss ICI therapy-induced side effects, and then introduce representative phytochemicals along with their advantages and shortcomings in cancer treatment. Finally, we comment on combination therapies of immune checkpoint inhibitors with phytochemicals, with a focus on the future development of this novel anti-cancer therapy.

## Immune checkpoints

Numerous immune checkpoint proteins, such as PD-1 (programmed cell death protein 1)/ PD-L1 (programmed cell death ligand 1) and CTLA-4 (cytotoxic T lymphocyte-associated antigen-4), are involved in tumor proliferation, angiogenesis, metastasis and chemoresistance *via* suppressing cytotoxic T cell activation, resulting in cancer immune escape and immune tolerance 39, 40. Relevant laboratory or clinical studies and public interest in ICI therapy keep increasing; here we introduce five well-studied immune checkpoints and their influence in cancer development.

### PD-1

PD-1 is a member of the CD28 superfamily expressed on CD4 and CD8 T cells, B cells, monocytes, natural killer (NK) cells, and dendritic cells (DCs) 41, 42. PD-1 interacts with its two ligands, PD-L1 or PD-L2, to deliver negative signals and exert multiple immunoregulatory roles in T cell activation and tolerance 43. PD-L1 is expressed on many cancer 44, and, on the other hand, PD-L2 is mostly restricted to activated DCs and macrophages 45. Interactions of PD-1 with PD-L1/PD-L2 have been reported in a wide variety of solid tumors and hematologic malignancies, with patients having tumors positive for PD-L1 or PD-L2 showing dramatically lower survival rates than those having tumors negative for both of these ligands (46% vs. 83% for 5-year survival) 46.

Currently, the modulatory effects of PD-1 on tumor growth and on immunity have been extensively studied, leading to the development of targeting antibodies such as pembrolizumab, nivolumab, cemiplimab, etc. 47, 48. However, a large proportion of patients do not respond to such treatments, which, additionally, may cause various immune-related side effects 49, calling for further studies to elucidate the involved mechanisms and relevant biomarkers for patient selection.

### CTLA-4

CTLA-4 is a homolog of CD28 expressed on activated T cells and Treg cells. It binds to B7 molecules with high affinity and negatively regulates immune responses 50. Although CD28 also binds to B7 molecules and co-stimulates T cells together with the T cell receptor (TCR), CTLA-4 has higher affinity for B7-1 (CD80) and B7-2 (CD86) and hence can outcompete CD28, leading to reduced release of pro-effector cytokines such as IL-12 and cytotoxic enzymes 51. As one of the most extensively studied immune checkpoints, CTLA-4 has been the target of inhibitor development, with ipilimumab being the first to be approved by FDA in 2011, for attacking melanoma cells 7. Currently, anti-CTLA-4 monoclonal antibodies (mAbs) are widely used in the treatment of multiple solid cancers, including melanoma, renal cell carcinoma, and colorectal cancer. Due to adverse side effects and limited observation, other CTLA-4 blockades that have shown good anti-cancer effects in many trials, such as tremelimumab and nivolumab, have not received FDA approval. Additionally, the combined use of anti-CTLA-4 mAbs and other anti-cancer molecules has shown positive outcomes, although further work is needed regarding potential adverse effects of such applications 52.

### LAG-3

LAG-3 (Lymphocyte activation gene 3) is one of the immune inhibitory receptors (IRs), expressed on activated T cells, Treg cells and NK cells, and is upregulated by IL-2, IL-7, IL-10 and IL-12. Major histocompatibility complex class II (MHC-II), LSECtin and Galectin-3 are representative ligands of LAG-3 53. The expression of LAG-3 and MHC-II may be coordinated in regulating T-cell-mediated immune responses. While the binding of these two activation antigens negatively regulates activated T cells, LAG-3 alone may work as a negative regulator to prevent the exacerbation of a variety of autoimmunity diseases, and has expected to be a promising therapeutic target in autoimmune diseases 54, 55. Recent studies support the view that blockade of LAG-3 significantly enhanced antitumor immunity, and its combination with other immune checkpoint inhibitors remarkably improves the efficacy of antitumor therapy 56.

### Tim-3

Tim-3, a member of the novel T cell immunoglobulin and mucin domain (Tim) family originally identified as a specific marker for Th1 and Tc1 cells, is also expressed on Treg cells, NK cells, monocytes, macrophages, and DCs 5. Upon interaction with a ligand, Tim-3 suppresses the immune responses, facilitating the development of diseases. As a negative immune regulator, Tim-3 is implicated for a role in autoimmune diseases. Galectin-9 has been confirmed as a classical ligand for Tim-3 and their binding may cause Th1 cell death and induce immune tolerance in tumor microenvironment 57. Additionally, several clinical trials have demonstrated that the expression of Tim-3 is associated with severe dysfunction of T cells in different types of cancers such as non-small-cell lung carcinoma (NSCLC), renal cell carcinoma (RCC), colon cancer and gastric cancer 58, 59. Indeed, on exhausted CD8+ T cells isolated from patients infected with virus, Tim-3 expression is often upregulated, which is probably responsible for inefficient antiviral activity 60. However, growing evidence indicates that, upon binding with Galectin-9, Tim-3 helps reduce tissue inflammation and hence suppress autoimmunity 61.

### TIGIT

TIGIT (T cell immunoglobulin and ITIM domain) is an inhibitory receptor expressed on the surface of NK cells, Treg cells, follicular T helper cells and subsets of regulatory and memory CD4+ and CD8+ T cells 62. TIGIT possesses a single extracellular immunoglobulin variable (IgV) domain that is responsible for binding nectin-2 (CD112), nectin-3 (CD113) and necl-5 (CD155). TIGIT interacts with CD155 expressed on antigen-presenting cells or tumor cells to downregulate T cell and NK cell functions, resulting in T cell suppression and the limitation of NK cell activation 63. TIGIT up-regulation has been observed in various malignancies, including melanoma, breast cancer, non-small-cell lung carcinoma (NSCLC); indeed TIGIT expression on peripheral blood CD8+ T cells of various cancer patients has been associated with metastases and poor survival 64. Solid tumors are currently targeted with anti-TIGIT mAbs in various stages of clinical trials, and blockades of TIGIT, such as tiragolumab, BGB-A1217 and AB154, are ongoing. Of note, compared to either monotherapy, dual TIGIT blockades, especially when combined with other ICIs, are likely to be more effective and promising 65, 66.

## Immune checkpoint inhibitor therapy

Many compromised measures have been used for cancer treatment, such as surgery (much normal tissues have to be removed) and chemo- and radio-therapies (severe side effects including damage to immune and hemopoietic systems), but cure or even alleviation is often hardly achievable. This situation calls for novel strategies for cancer treatment. Among the newly developed anti-cancer strategies ICI therapy are promising research direction. Many of immune checkpoint blockades are under assessment in clinical trials or have been approved by the FDA. Below, we summarize some representative inhibitors of immune checkpoints - their effectiveness and application, along with their adverse effects (Table 3).

### PD-1/PD-L1 inhibition

In the past few years, inhibitors of PD-1 and its ligand PD-L1 are increasingly used for the treatment of selected cancer patients and are highly effective in many cases. Nivolumab (MDX-1106 or BMS-936558), an antibody targeting PD-1, has been approved by FDA for the treatment of a broad panel of malignancies, including advanced NSCLC, melanoma, classical Hodgkin lymphoma, squamous cell carcinoma of the head and neck (HNSCC), renal cell carcinoma (RCC), urothelial carcinoma (UC), hepatocellular carcinoma (HCC), and colorectal cancer (CRC) 8, 67, 68. In a Phase I trial of nivolumab, patients with melanoma, renal cell carcinoma and NSCLC had response rates of 28%, 27% and 17%, respectively 69.

Pidilizumab (CT-011) is the first PD-1-targeting humanized mAb to be tested in clinical trials for melanoma, NSCLC, renal cell carcinoma, head and neck cancers, lymphoma and several other cancers 70. Other PD-1 inhibitors encompass pembrolizumab (MK-3475, lambrolizumab) and cemiplimab, which respectively exhibit acceptable tolerance and remarkable efficacy in several tumors such as advanced NSCLC and advanced squamous cell carcinoma (SCC) 14.

Atezolizumab (MPDL3280A) inhibits the PD-L1/PD-1 pathway by binding to PD-L1 and has been approved for the treatment of advanced NSCLC, triple-negative breast cancer and urothelial carcinoma 15, 71. Another humanized PD-L1 monoclonal antibody durvalumab (MEDI4736) with T cell dependent anti-tumor activity shows efficacy in the treatment of patients with urothelial carcinoma 72.

### CTLA-4 inhibition

Ipilimumab (MDX-010, Yervoy; Bristol-Myers Squibb) is a fully human monoclonal antibody against CTLA-4 (cytotoxic T-lymphocyte antigen-4) for the treatment of melanoma patients and was approved by FDA in 2011 7. Over the past decade, ipilimumab has demonstrated efficacy in the treatment of renal cell cancer (RCC), lung cancer, metastatic melanoma and prostate cancer in addition to melanoma 73, 74. In a series of trials on combined applications of immune checkpoint inhibitors in cancer treatment, cohort A (nivolumab plus ipilimumab) demonstrated better treatment outcomes than cohort B (nivolumab alone), as judged by reduced tumor metastases and effective extension of progression-free survival. Unfortunately, however, these improvements were not of significance and in many cases this combined therapy resulted in more serious side effects 75.

Melanoma patients with ipilimumab monotherapy showed excellent overall response rates, with survival time of many patients being significantly prolonged 76. In a phase 2 dose-ranging study, patients with pretreated advanced melanoma were randomly assigned a fixed dose of ipilimumab of either 10 mg/kg (n=73), 3 mg/kg (n=72), or 0.3 mg/kg (n=72) every 3 weeks for four cycles, followed by maintenance therapy every 3 months. The best overall response rate was 11.1% (95% CI 4.9-20.7), 4.2% (0.9-11.7), and 0% (0.0-4.9) respectively. Of note, in the trial, some patients showed immune-related adverse events (irAEs) 77. Nevertheless, considering the favorable clinical outcomes, National Comprehensive Cancer Network (NCCN) has added ipilimumab as a category 1 recommendation in the guidelines of systemic therapy options for advanced or metastatic melanoma. The irAEs, such as enterocolitis, rash, hepatitis, hypophysitis, uveitis, pancreatitis and leucopenia, could be rather serious, so it is critical to carefully select the patients and use ipilimumab at appropriate stages for optimal treatment.

Tremelimumab (CP-675206) is also a humanized monoclonal antibody specific for treatment of cancers such as malignant mesothelioma 78, mostly as an adjuvant agent with other mAbs 79.

## Measures against ICI related adverse effects - combined use of phytochemicals

Immune-related adverse events most commonly involve the gastrointestinal tract, endocrine glands, skin, liver, and the nervous and endocrine systems 80. Common symptoms include diarrhea, rash, muscle weakness, headache, fatigue and other minor discomfort, which are not fatal but affect patients' quality of life 81 (Table 3). The precise pathophysiology underlying the immune-related adverse events is under scrutiny, and different blockades of immune checkpoints may cause different adverse effects on patients with different or even histologically indistinguishable cancers, often presenting a dilemma for the physician to decide whether to use ICI. However, the prestigious efficacy of ICI calls for measures to make full use of this special anticancer strategy and in the meantime mitigate its side effects. One way is to combine ICI therapies with phytochemicals.

Owing to their ubiquitous availability and special biological activities, often of great medical significance, plant-derived natural compounds have had tremendous impacts on drug discovery and continuously receive FDA approval 82. Phytochemicals can be divided into multiple groups, such as polyphenols, terpenes, alkaloids and others, and polyphenols can be classified into flavonoids (e.g., apigenin and EGCG) and no-flavonoids (e.g., resveratrol and curcumin). We previously have demonstrated the efficacy of plant materials against diseases including malignancies and the beneficial effects of these natural products could be attributed to the phytochemicals such as lignans 23, 24, 83-85. According to previous studies, the combination of phytochemicals with standard chemotherapeutic drugs could significantly improve cancer patient survival 86-88. Furthermore, phytochemicals contribute to the balance of intestinal microbiota, increasing beneficial microbial metabolites, thus exerting anti-tumor effects and helping improve the efficacy of immunotherapy 89, 90 (Figure 4).

**Figure 2:**
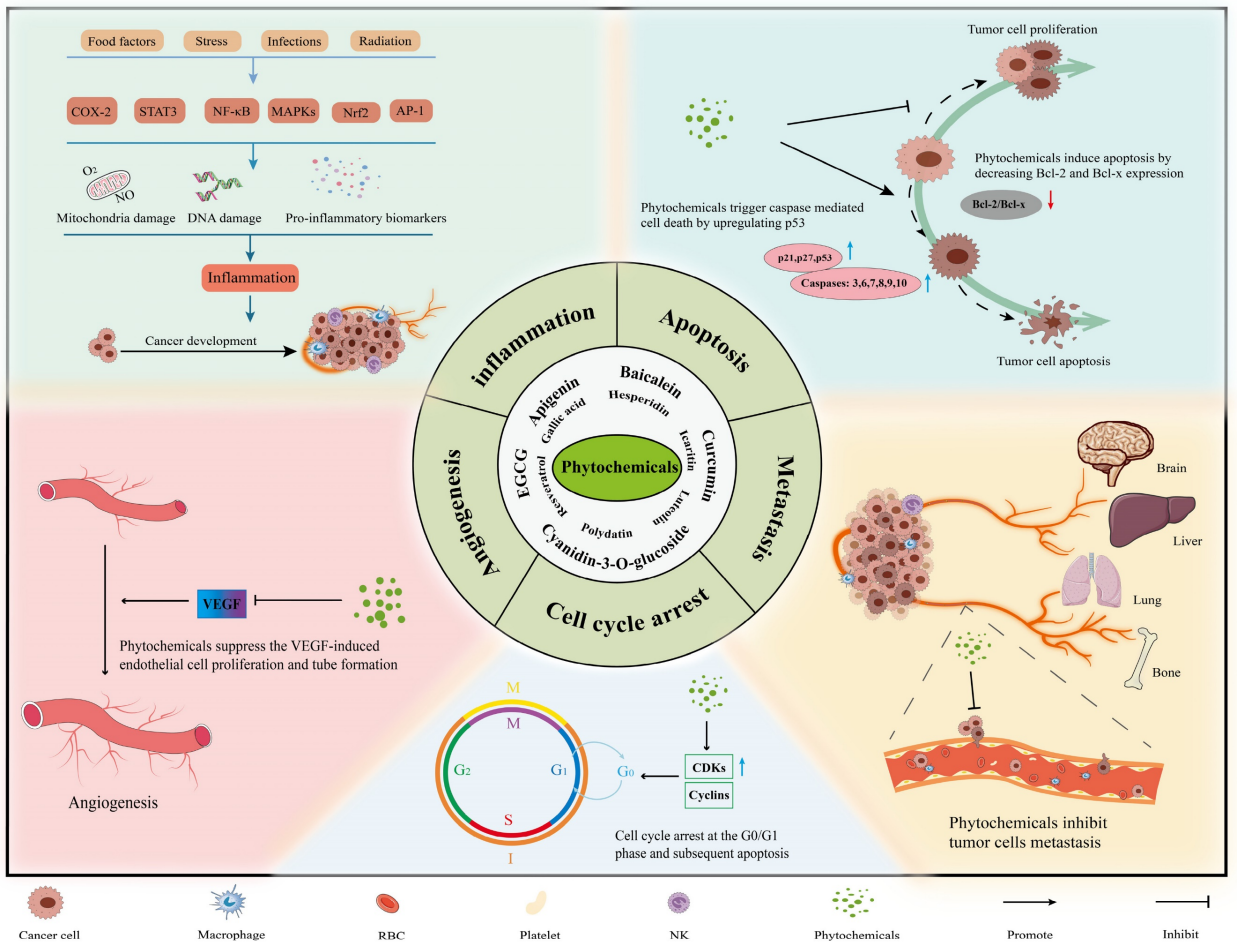
** The main mechanisms and effects of phytochemicals against tumors.** Most phytochemicals participate in tumor suppression by inhibiting tumor metastasis, angiogenesis and inducing tumor cell apoptosis and cell cycle arrest.

**Figure 3:**
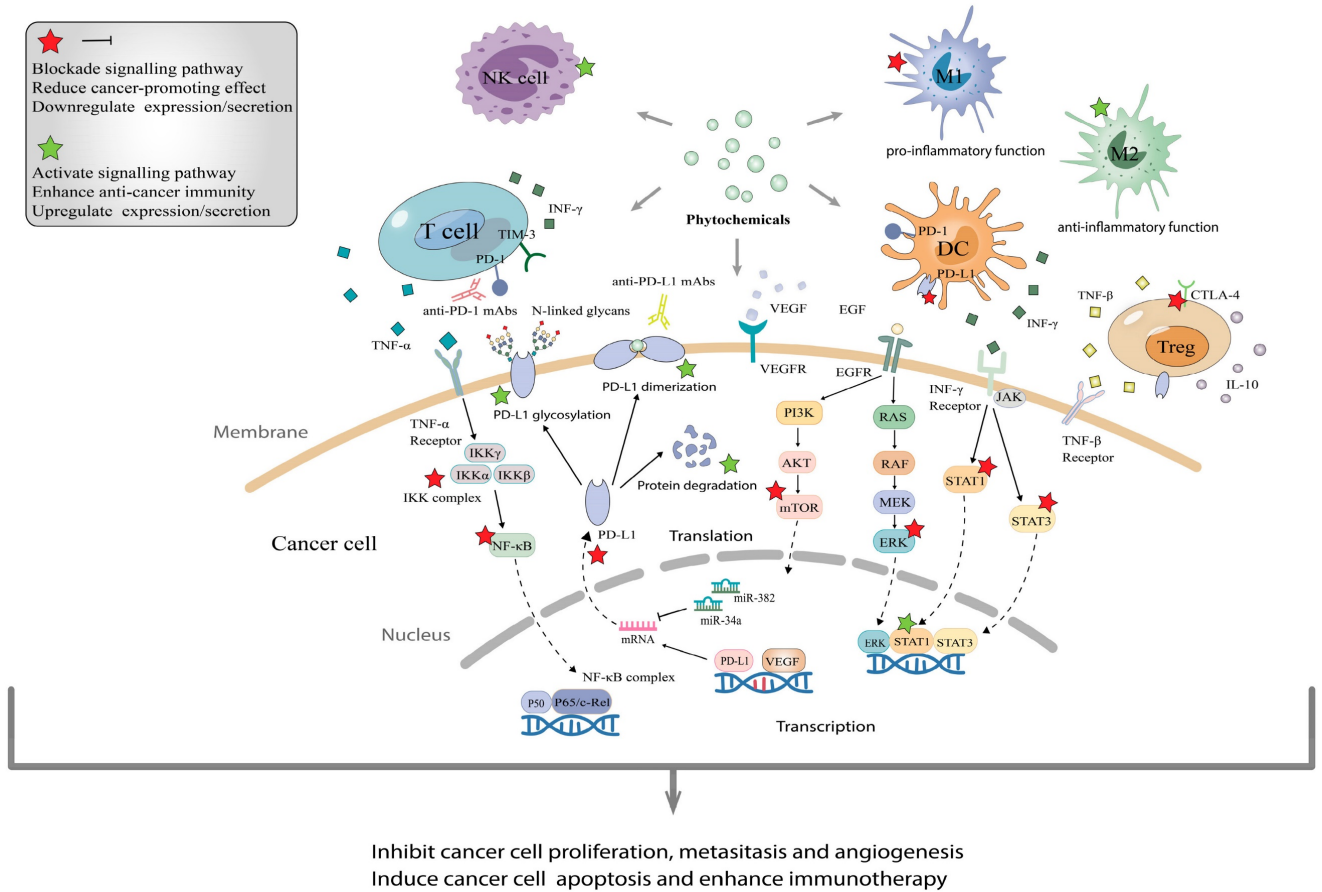
** The molecular mechanisms of phytochemicals in combined application with immune checkpoint inhibitors.** (A), Phytochemicals usually enhance immune checkpoint inhibitor therapy *via* activating immune cells that recognize and kill cancer cells such as T cells, NK cells and DC, as well as suppressing pro-inflammatory and carcinogenetic function of immunosuppressive cells. (B), Phytochemicals block the interaction between PD-1 and PD-L1 by decreasing the expression and promoting dimerization/glycosylation of PD-L1, even facilitating their degradation. (C), Through blocking various signaling pathways such as PI3K/AKT/mTOR, JAK/STAT3 and VEGF, phytochemicals help ICIs to inhibit tumor proliferation, metastasis and angiogenesis, then induce apoptosis.

Phytochemicals fight against tumors mainly through inducing apoptosis of cancer cells, facilitating cell cycle arrest, disabling immunosuppressive Tregs and inhibiting neoangiogenesis (Figure 2). In experiments with the combined application of phytochemicals and immune checkpoint inhibitors, we found that phytochemicals promote the efficacy of ICIs by down-regulating the expression of immune checkpoints or/and their ligands (e.g., PD-1/PD-L1), as well as by blocking the pathways associated with cancer progression (e.g., PI3K/AKT, EGFR) (Figure 3). In addition, they can also affect drug metabolism by regulating intestinal microbiota 84. Below are representative phytochemicals that have significantly enhanced cancer treatments alone or combined with ICI therapies.

### Resveratrol

Resveratrol, richly present in a variety of plants such as grapevine, peanut and pine, exhibits potent anti-inflammatory, antioxidant, anti-viral activities and has demonstrated therapeutic properties against several cancers including breast, gastric, lung, prostate and thyroid cancers 91, 92. Resveratrol inhibits the growth and development of tumors through nuclear factor-κB (NF-κB) signaling. Activation of nuclear factor-κB (NF-κB) often occurs in tumor cells and contributes to aggressive tumor growth and resistance to chemotherapy and radiotherapy, whereas resveratrol may downregulate NF-κB and thus increase the therapeutic efficacy by lowering the threshold for tumor cell apoptosis 93. Of significant importance, resveratrol may enhance the effects of ICI therapy by modulating the expression of immune checkpoint and its ligand, PD-1, PD-L1 and CTLA-4, and hence mitigate immune-related adverse effects caused by ICIs. Indeed, the combined use of resveratrol and PD-1 antibody could greatly inhibit tumor growth in ovarian carcinoma, while anti-CD8 antibody co-treatment would restore the tumor growth 94. Verdura et al. reported that resveratrol can inhibit glyco-PD-L1-processing enzymes (α-glucosidase/α-mannosidase) that modulate N-linked glycan decoration of PD-L1, thereby promoting the endoplasmic reticulum retention of a mannose-rich, abnormally glycosylated form of PD-L1, ultimately impeding its targeting to the cancer cell membrane and promoting immunity 95. These well-conducted experiments and the unforeseen immunomodulating mechanisms by resveratrol might illuminate new approaches to restore T-cell function by targeting the interaction of PD-1 and PD-L1. It is worth mentioning that resveratrol has a boosting effect on gut microbiota, the microbiota can modulate multiple cellular events such as cellular metabolism and host immune function to regulate internal homeostasis and regress the development of virous cancers 96.

### Curcumin

Curcumin is an active component of the dietary spice turmeric that has been widely used for treatment of illnesses in Asian countries, such as China and India, and some European regions 97. Over several centuries, curcumin has been a key ingredient in many traditional medical recipes for treatment of a large variety of diseases, including digestive disorders, common infections, dermatologic diseases and depressive states 98.

As a kind of phytochemicals, curcumin has many well documented bioactive effects, including anti-inflammatory, anti-oxidative, immune-protective, metabolic stability and anti-tumor functions and, therefore, may potentially be used in the treatment of diseases such as cancer therapy 97. Many lines of evidence demonstrate that curcumin inhibits tumor progression by modulating multiple pathways (e.g., NF-κB, MAPK, STAT3; 99), coordinating tumor suppressor factors (e.g., P53, cytokines IL-1, -2, -6, -8, or -12, IFN-γ) to promote cancer cell apoptosis and clearance 100, 101, and inhibiting angiogenesis 102. Curcumin, used alone or combined with other treatments, has shown robust effects in suppressing the progression and metastasis of cancers, such as tongue squamous cell carcinoma, prostate cancer, pancreatic cancer, breast cancer and colorectal cancer, and, especially, in enhancing the response of immune therapy 103. When combined with immune checkpoint inhibitors such as anti-PD-1/PD-L1 and anti-CTLA-4 mAbs, curcumin presented excellent antitumor effects by downregulating cytokine secretion, inhibiting NF-κB pathway and promoting the infiltration of anti-tumor T cells 101. Analogs of curcumin, e.g., bisdemethoxycurcumin, in combination with an anti-PD-L1 antibody, may significantly boost immune responses and prolong survival in animal experiments 104. Hayakawa et al. combined curcumin with PD-1/PD-L1 Abs in MC38 murine tumor models and achieved synergistic anti-tumor effects and induced tumor antigen-specific T cells, significantly augmenting the efficacy of treatment 105. In addition to showing excellent results in the cell lines and animal models, the combined application of curcumin and ICIs is also beginning to be studied in some clinical trials, accelerating their clinical use in cancer patients 106. In this trial, patients obtained good therapeutic results in cervical and uterine cancer by combining PD-1 blockades Pembrolizumab with curcumin as a dietary supplement. Additionally, researchers think the alterations in gut microbiota could contribute to identify mechanisms of therapy, resulting in predictive biomarkers for efficacy and improved patient selection in future clinical applications.

### EGCG (epigallocatechin-3-gallate)

Catechins are bioactive constituents of tea with a variety of beneficial effects on human health and have long been the targets of active research for cancer prevention and therapy 107. EGCG (epigallocatechin-3-gallate) is among the most potent anti-cancer elements in tea and is widely used in the treatment of cancer and many other diseases, alone or with traditional or ICI cancer therapies to reduce side effects 108, 109. Its antitumor mechanisms encompass inducing cell cycle arrest, apoptosis and autophagic cell death, and it can reduce cisplatin-induced ototoxicity, DOX-induced cardiotoxicity, and cisplatin-induced neurotoxicity via inhibiting the STAT1 (signal-transducer-and-activator-of-transcription-1) and NF-κB signaling pathways 110. EGCG could suppress lung tumor growth by inhibiting PD-L1 expression through numerous pathways, such as JAK2/STAT1 signaling and EGFR/Akt signaling, and therefore significantly restore T cell function 26. While there are limited examples of the combination of EGCG and ICIs, a previous study has reported that EGCG may inhibit PD-1/PD-L1 interactions by dimerizing PD-L1. Through utilizing molecular modeling and computer analysis, researchers illustrate how EGCG induce and stabilize PD-L1 dimerization, highlighting its potential as a small anti-cancer molecule or complementary therapeutic drug in cancer immunotherapy 111. However, further experiments verification is demanded to provide more comprehensive understandings on the inhibitory mechanism of EGCG in anti-PD-1/PD-L1 treatment, especially regarding its combination with immune checkpoint inhibitors.

### Remaining questions and promising solutions about phytochemicals

**Figure 4:**
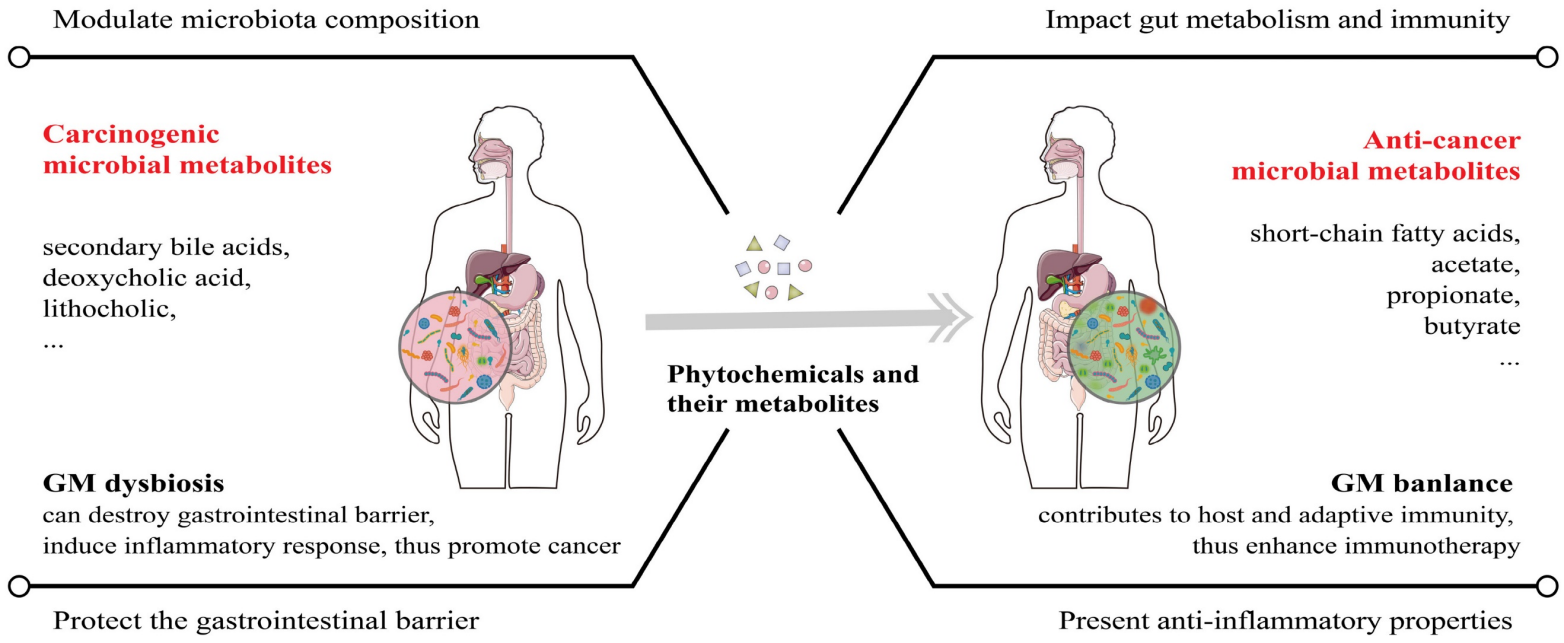
** The regulation function of phytochemicals in GM (gut microbiota) balance and potential anticancer effects.** GM dysbiosis is a major cause of cancer, both within and outside the gastrointestinal tract. Phytochemicals and their active metabolites can regulate GM balance to repair the gastrointestinal barrier and strengthen the host immunity, thus exerting cancer suppressive effects and enhancing ICI therapy efficacy.

The greatest advantage of phytochemicals is their potent anti-tumor properties with low toxicity, ideal for effective and safer cancer treatment. They suppress cancer cells mostly by modulating the expression of immune checkpoints or their ligands, activating T cells, inducing cell cycle arrest and apoptosis, and cooperating with general defense mechanisms of the human body such as gut microbiota and the innate immune functions 24, 112, 113 (Figure 2-4), rather than simply and directly killing cancer cells, resistance by the targeted cancer cells can maximally be reduced or avoided. Additionally, the majority of phytochemicals used in cancer treatment possess anti-oxidative, anti-inflammatory, or immune-protective properties, thus they can protect normal tissues from chemotherapy- and radiotherapy-induced toxicity and therefore mitigate related side effects. Collectively, phytochemicals have a great potential to be a very effective adjuvant to help improve the effectiveness of other anti-cancer treatments including traditional therapy and novel immunotherapy.

Unfortunately, phytochemicals, though ubiquitous and richly present in dietary plants, usually do not have the required amounts sufficient for cancer treatment or prevention. Furthermore, they may not stay long enough in the body to exert the desired anti-cancer functions. Many of them may have low water solubility and poor absorption (when phytochemicals reach the intestinal tract, they are rapidly metabolized), leading to low bioavailability to the cancer cells 114, 115. Aim to increase the dispersibility, stability and bioactivity of phytochemicals, several delivery systems including nanoparticles, liposomes, and micelles have been investigated. With the continuous development of nanotechnology, new nano-materials and nanotechnology are increasingly used in medicine and pharmacy 116. After the phytochemical is nano-sized, the physical properties (e.g., saturation solubility and hydrophilicity) and biological properties (e.g., molecular affinity) of it, are changed. For curcumin, low bioavailability is a common problem with oral administration of it 117, curcumin oral nanoformulations show higher solubility in various trials, encouraging us to develop formulations used in clinic with higher bioavailability 118. On the other hand, not all phytochemicals are non-toxic, especially high dose of them should be used to meet effective dose, probably causing serious adverse effects such as allergic reactions, or liver or kidney toxicity, which is a topic awaiting systematic investigations.

As mentioned above, we found that many phytochemicals inhibit tumor proliferation by down-regulating PD-L1 expression, but some studies have shown that different phytochemicals have opposite effects on PD-L1 levels in different types of tumors, some even increase PD-L1 expression which may decrease the efficacy of the treatment 28, 119. At the same time, many observations, including our previously published article, have disclosed the relevance of phytochemicals in the abundance of beneficial bacteria with contributions to the health of human beings 84, 120. Furthermore, mounting evidence support that microbiome exhibit anti-cancer effects as a factor rather than a biomarker in assessing the efficacy of ICI therapy 121. Therefore, there is a great need to rationally conduct more experiments such as cellular lines and animal models to elucidate the anti-tumor mechanisms of the combined application of phytochemicals plus ICIs, which will provide sufficient rationale to facilitate precision medicine. Considering the pivotal role of phytochemicals on human gut microbiome, establishing corresponding antitumor models to analyze the connection between differential phytochemicals and the abundance of beneficial bacteria is feasible and promising, which will also broaden application prospects of phytochemicals.

## Combination therapy: conclusions and perspectives

In the current clinical setting, the main strategies to treat cancer still, like the last decades, consist of conventional methods including surgery, chemo- and radio-therapies, which do not provide adequate efficacy and often face recurrence, although novel therapies such as ICI therapy are becoming a choice. However, the low response of the disease to ICI and the high instance of adverse effects of ICI have seriously hampered this kind of treatment. Many patients receiving this therapy do not respond positively or experience immune-related adverse events (irAEs), such as gastrointestinal toxicity, endocrine toxicity, and dermatologic toxicity.

To overcome such problems, combination therapy comes to clinical application and provides great potential. Compared to monotherapies, combination therapy offers three major advantages, including considerably improved anti-cancer efficacy, expanded drug usage, and delayed drug resistance. The key approach to successful application of combination therapy is the selection of drugs based on the personalized medical information of the patient that act on different pathways or different mechanisms without cross-resistance or overlapping adverse effects.

In order to achieve good treatment results, reduce patient suffering and ease the economic burden, the continuous discovery and improvement of novel economical phytochemicals is a core issue. For example, lignans, such as enterolactone, enterodiol, secoisolariciresinol diglucoside or other compounds 24, 122, 123, have excellent suppressive effects on cancers alone, and we are in the process of evaluating these phytochemicals for combination therapy with ICI. To date, numerous experiments (e.g., *in vitro*, *in vivo*, clinical trials) has shown enormous potential of ICI plus phytochemicals combination in virous tumors, so it's hopeful to see their application in clinic in the near future 106, 124, 125. With the development of high-throughput sequencing technology and popularization of personalized therapy, precision medicine based on genomic analysis (e.g., Next Generation Sequencing) has been popular and approved for clinical application of various diseases including cancer 126, 127. In the meanwhile, gut microbiota genomic sequencing (e.g., 16S rRNA, whole genome sequencing) is also gradually applied in the field of cancer treatment, having a great prospect for choosing the appropriate treatment plan for patients and evaluating the treatment efficacy 128. Additionally, toxicity assessment is also critical to the development of phytochemicals to aid less side effects and better treatment efficacy, whether evaluating single agent or agents used in combination. And the studies in predictive biomarkers for differentiating responders and nonresponders will greatly help in patient selection and alleviate immune-related adverse events.

The application of ICI plus phytochemicals combination has a promising perspective, not only should we be optimistic about this therapy, but also face their shortcomings and obstacle in clinical use. As we sated above, elucidating the activity and potential toxicity of various phytochemicals in different tumors as well as designing the mode of administration to promote their biological activity are of vital interest to boost the combination with ICI therapy. Furthermore, in order to facilitate the development of personalized therapies and the progress of immunotherapy, we should pay more attention to the discovery of relevant indicators to measure the effectiveness of the combination therapy.

## Figures and Tables

**Figure 1:**
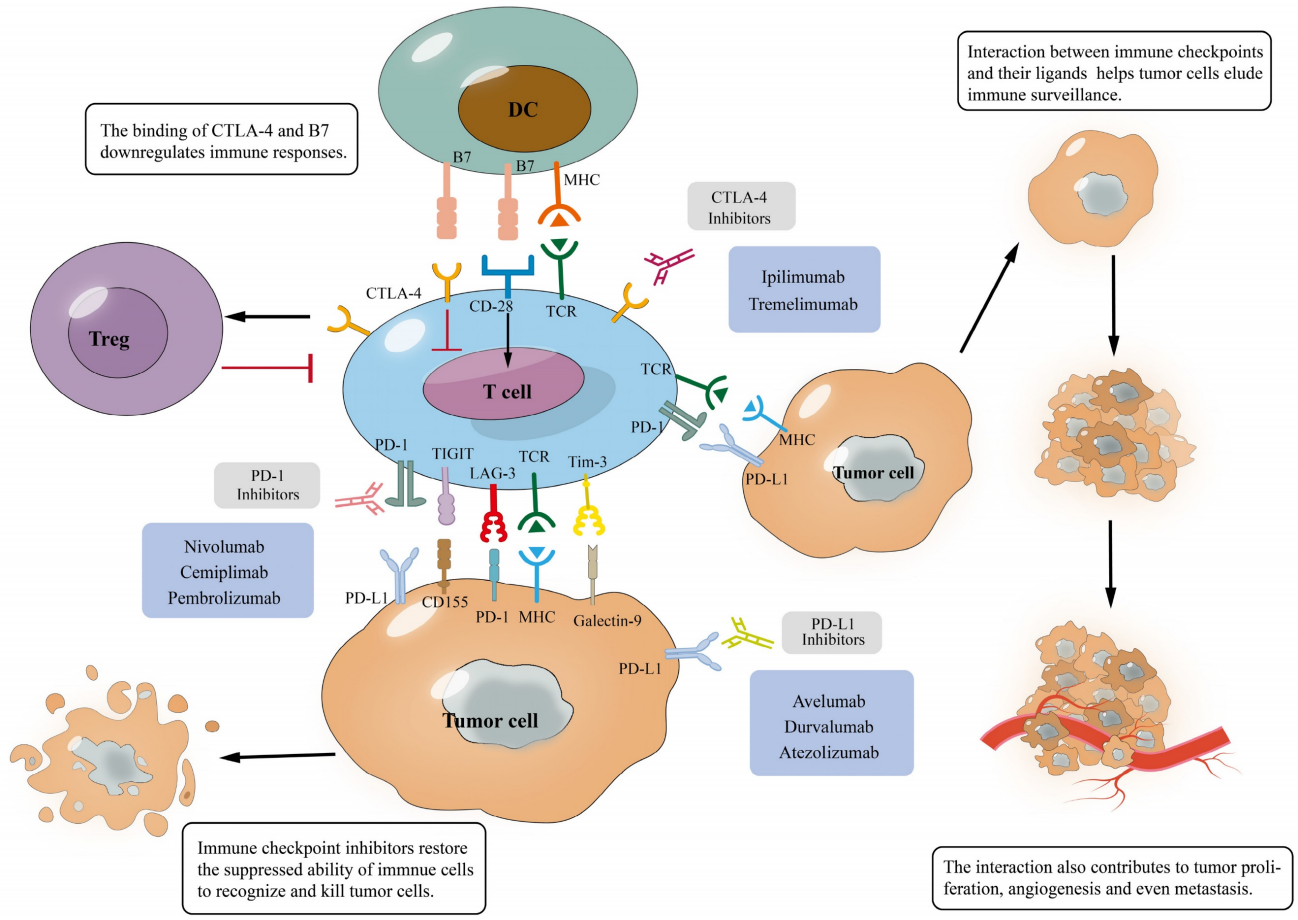
** Interaction of immune checkpoints with their ligands and mechanism of immune checkpoint blockades.** (A), The binding of immune checkpoints with their ligands can inhibit T cells and help tumor cells evade immune surveillance, which will promote tumor development; (B), The binding of the antibody to immune checkpoints prevents them from binding to ligands, inducing T cell activation and tumor cell apoptosis, which eventually improves anti-tumor immunity.

**Table 1:** Immune checkpoint inhibitors (ICIs) approved by FDA.

Agent	Target	Target cancer type	Ref
Ipilimumab	CTLA-4	Melanoma	7
Nivolumab	PD-1	Melanoma, NSCLC, SCLC, SCC, Urothelial carcinoma, Classic Hodgkin's lymphoma, RCC, HCC	8, 10
Pembrolizumab	PD-1	Melanoma, NSCLC, Gastric or gastroesophageal junction, Merkel cell carcinoma, RCC, HCC, head and neck SCC, Endometrial carcinoma	11-13
Cemiplimab	PD-1	Advanced SCC	14
Atezolizumab	PD-L1	Urothelial carcinoma, NSCLC, PD-L1 triple-negative breast cancer, SCLC, Melanoma, HCC	15, 16
Durvalumab	PD-L1	Urothelial carcinoma, NSCLC, SCLC	17
Avelumab	PD-L1	Merkel cell carcinoma, RCC, Urothelial carcinoma	18, 19

**Table 2:** List of phytochemicals and their effects on PD-L1 regulation in several anti-cancer studies.

Classification	Plant Origin	Compound	Effect	Molecule mechanism	Cancer type (model)	Ref
Flavonoids	Matricaria chamomilla	Apigenin	PD-L1 ↓	Inhibits PD-L1 expression via IFN-γ-induced STAT1 activation	Breast cancer (cell lines)	27
	Scutellaria baicalensis	Baicalein	PD-L1 ↓	Downregulates PD-L1 expression via inhition of STAT3	Hepatocellular carcinoma (mouse model)	28
		Baicalin				
	Arbutus unedo	Cyanidin-3-O- glucoside	PD-1 and PD-L1 ↓	Inhibits VEGF and PD-L1 expression	Colon cancer (cell lines)	31
	Dictamnus dasycarpus	EGCG (Epigallocate-chin gallate)	PD-L1 ↓	Reduces IFN-γ-induced PD-L1 expression via inhibition of JAK2/STAT1 signaling and decreases EGF-induced PD-L1 expression through inhibition of EGFR/Akt signaling	Lung cancer, mouse melanoma (mouse model)	26
	Citrus species	Hesperidin	PD-L1 ↓	Suppresses Akt and NF-κB signaling and induces PD-L1 downregulation	Breast cancer, oral cancer (cell lines)	33, 129
	Epimedium koreanum	Icaritin	Synergistic effect with anti-PD-1 Ab ↓ PD-L1 ↓	Blocks IKK complex formation and NF-κB translocation which promote PD-L1 expression	Liver cancer (clinical trial)	34
	Matricaria chamomilla	Luteolin	PD-L1 ↓	Inhibits the IFN-γ dependent PD-L1 upregulation	Breast cancer (cell lines)	27
Non-flavonoids	Curcuma longa	Curcumin	PD-L1 ↓	Inhibits STAT3 pathway and reduces Treg (CD4+CD25+FOXP3+) and MDSC	Tongue squamous cell carcinoma (cell lines and mouse model) external cancerous lesions (clinical trial)	29, 130
	Camellia sinensis	Gallic acid	PD-L1 ↓	Suppresses EGF binding on EGFR resulting PI3K/AKT pathway inhibition, upregulates p53 and miR-34a and downregulates PD-L1 expression	Lung cancer (cell lines)	32
	Polygonum cuspidatum	Polydatin	PD-L1 ↓	Enhances miR-382 and inhibits miR-382-induced PD-L1 expression	Colon cancer (cell lines)	35
	Vitis vinifera	Resveratrol	PD-L1 ↓	Inhibits thyroxine-induced PD-L1 expression	Oral cancer (cell lines)	25
				Inhibits PD-L1 Glycosylation and Dimerization	Breast cancer (cell lines)	119
Terpenes	Epimedium koreanum	β-Elemene	PD-L1 ↓	Regulates AKT signaling, thereby controlling the expression of PD-L1	Esophageal cancer (cell lines and mouse model)	131
	Panax ginseng	Ginsenoside Rg3	PD-L1 ↓, CD8+T↑	Suppresses Akt and NF-κB signaling and induces PD-L1 downregulation, as well as promote CD8+T cells	Lung cancer (cell lines)	132
		Ginsenoside Rh2	PD-L1 ↓	Suppresses PI3K-Akt and EGFR signalings and induces PD-L1 downregulation	Lung cancer (cell lines)	133
		Ginsenoside Rk1	PD-L1 ↓	Suppresses NF-κB signaling and induces PD-L1 downregulation	Lung adenocarcinoma normal lung epithelial cell (mouse model)	134
	Platycodon grandiflorus	Platycodin D	PD-L1 ↓	Reduces the level of PD-L1 in lung cancer cells via triggering its release into the cell culture medium	Lung cancer (cell lines)	135
Alkaloids	Berberis plants	Berberine	PD-L1 ↓	Enhances the sensitivity of tumor cells to co-cultured T-cells by decreasing the level of PD-L1 in cancer cells	Non-small-cell lung cancer (NSCLC) (mouse model)	136
	Ecteinascidia turbinate	Trabectedin	blocks the PD-1/PD-L1 axis	Modulates transcription and translation of IL6, CCL2, and IFNα in myeloid cells and FOXP3 in regulatory T cells, also blocks the PD-1/PD-L1 axis by targeting PD-L1+ CLL cells, PD-L1+ monocytes/macrophages, and PD-1+ T cells	Chronic lymphocytic leukemia (CLL) (mouse model)	137
Others	Chaenomeles speciosa	Ethanol extract (EEC)	PD-L1, Foxp3, TGF-β ↓	Inhibits the expression of PD-L1, Foxp3 and TGF-β to suppress tumor growth	Hepatoma (cell line)	138
	Sarcodon imbricatus	Aqueous extract	IL-6↑ PD-L1↓	Increases serum concentrations of IL-2, IL-6 and tumor necrosis factor-α, natural killer cell activity and the viability of splenocytes and reduces the expression of PD-L1	Breast cancer (mouse model)	139

**Table 3:** Common immune-related adverse effects.

Affected area	Symptoms
Gastrointestinal system	Diarrhea, abdominal pain, nausea, bowel perforations
Skin	Rash, pruritus
Liver	Right upper quadrant abdominal pain, nausea, vomiting
Nervous system	Muscle weakness, sensory neuropathies
Endocrine system	Headache, visual-field defects, fatigue, weakness, asthenia, nausea and vomiting, fever, hypotension, behavioral changes

## Abbreviations

CDK: cyclin dependent kinase
CRC: colorectal cancer
CTLA-4: cytotoxic T-lymphocyte-associated antigen 4
DC: dendritic cells
EGCG: epigallocatechin-3-gallate
ELAM-1: endothelial leukocyte adhesion molecule-1
FDA: U.S. Food and Drug Administration
HCC: hepatocellular carcinoma
HNSCC: head and neck squamous cell carcinoma
ICAM-1: intracellular adhesion molecule-1
ICI: immune checkpoint inhibitor
IFN-g: interferon-g
IL-1: interleukin-1
IL-2: interleukin-2
IL-6: interleukin-6
IL-7: interleukin-7
IL-8: interleukin-8
Il-10: interleukin-10
IL-12: interleukin-12
irAEs: immune-related adverse events
IRs: inhibitory receptors
JAK2: Janus kinase 2
LAG-3: Lymphocyte activation gene 3
lgV: immunoglobulin variable
mAb: monoclonal antibody
MAPK: mitogen-activated protein kinase
MHC-II: Major histocompatibility complex class II
NCCN: National Comprehensive Cancer Network
NF-κB: nuclear factor-κB
NK cell: natural killer cell
NSCLC: non-small-cell lung carcinoma
PD-1: programmed cell death protein 1
PD-L1: programmed cell death-Ligand 1
PD-L2: programmed cell death-Ligand 2
RCC: renal cell carcinoma
STAT-1: signal-transducer-and-activator-of-transcription-1
STAT3: signal-transducer-and-activator-of-transcription-3
TIGIT: T cell immunoglobulin and ITIM domain
Tim-3: T cell immunoglobulin and mucin domain-containing protein 3
Tregs: regulatory T cells
TSCC: tongue squamous cell carcinoma
UC: urothelial carcinoma
VCAM-1: vascular cell adhesion molecule-1
